# Treatment of Fatty Acid Oxidation Disorders Today: Emerging Personalized Treatment Strategies

**DOI:** 10.1002/jimd.70255

**Published:** 2026-09-25

**Authors:** U. Spiekerkoetter, J. Vockley

**Affiliations:** ^1^ Department of Pediatrics, Adolescent Medicine and Neonatology University Children's Hospital and Faculty of Medicine, Albert Ludwigs University Freiburg Germany; ^2^ UPMC Children's Hospital of Pittsburgh and University of Pittsburgh School of Medicine Pittsburgh USA

## Abstract

The first international guideline on treatment and management of long‐chain fatty acid oxidation disorders has been published and demonstrates little scientific evidence for many of the treatment decisions. However, it is a substantial achievement to harmonize treatment and to define the remaining challenges. We here deliver some thoughts on this guideline and highlight the tremendous effort to reach this international consensus.

## 1 Introduction

Fatty acid oxidation disorders (FAODs) are relatively newly recognized, with the first being identified in the 1970s. They are now known to have a high incidence (in total, 1:7000–10 000), particularly in populations of European origin. Many countries have newborn screening programs for these disorders [1, 2], thereby increasing the apparent disease frequency. While our understanding of the pathophysiology of long‐chain fatty acid oxidation disorders (LC‐FAOD) has significantly increased in the last two decades [3, 4], there have been few advances in treatments.

In 1950, the activation of carboxylic acids as part of the metabolic degradation of fatty acids in cells was characterized by Feodor Felix Konrad Lynen, a German biochemist at Munich University. A little more than 20 years later, the first inborn error of this pathway, carnitine palmitoyltransferase 2 deficiency, was identified based on metabolite patterns in patient samples. Characterization of primary carnitine deficiency and medium‐chain acyl‐CoA dehydrogenase (MCAD) deficiency soon followed; however, definitive confirmation of these entities by enzymatic and molecular analysis lagged by a decade or two. Since then, approximately 20 primary or secondary FAODs have been identified, most involving the metabolism of long‐chain fatty acids. The most recent conditions, recognized in the 2010s, were disorders of riboflavin transport and intracellular riboflavin metabolism to FAD [5, 6], which is an important cofactor of the fatty acid oxidation pathway, initially discovered in the 1930s.

C. Vianey‐Saban et al. published an article in 2023 in the Journal of Inherited Metabolic Disease (JIMD) entitled: *Fifty years of research on mitochondrial fatty acid oxidation disorders: The remaining challenges*. This publication provided a comprehensive overview of mitochondrial fatty acid oxidation defects and an extensive review of their clinical and biochemical phenotypes [7]. While most share similar clinical signs, largely due to energy depletion and toxicity of accumulating substrates, disease‐specific clinical symptoms exist. Moreover, a wide disease spectrum, from mild to severe, has been described in all. Especially after the introduction of newborn screening, individuals were identified who remained asymptomatic into adolescence and adulthood, who expressed a biochemical phenotype but had little likelihood of developing clinical disease. As molecular testing in patients became more routine, genotype–phenotype relationships sometimes became clear, though in some cases, patients with identical genotypes presented differently, presumably due to additional genetic and/or environmental modifiers. As a result, treatment standardization has been driven by local practice and experience rather than clinical trials. Perhaps of greatest concern was the realization that in the first years after the introduction of newborn screening, many individuals were burdened by unnecessarily strict dietary interventions. Subsequent long‐term follow‐up of such children allowed dietary restrictions to be relieved, with individualized treatment protocols. However, this has also led to heterogeneous treatment recommendations based on expert opinion across countries [8] and even between centers within a single country. To date, few standardized clinical trials have been performed, leaving the majority of treatment decisions without rigorous evidence.

## 2 The International Guideline on LC‐FAODs Based on Scientific Evidence and Expert Consensus to Harmonize Treatment Worldwide

To develop an international consensus on state‐of‐the‐art treatment and management, a guideline process was initiated in 2019, supported by the SSIEM and conducted in collaboration with the International Network for Fatty Acid Oxidation Research and Management (INFORM) and the European Reference Network for Hereditary Metabolic Disorders (MetabERN). Long‐standing international collaborations in the field of LC‐FAODs have prepared the ground for this international guideline. In particular, the collaboration within INFORM, founded in 2013 by doctors and researchers working in the field of fatty acid oxidation, promoted this initiative. INFORM is a global scientific organization that focuses on FAODs and related enzymatic disorders with the goal of advancing research and education to enhance best clinical practice. The network is open to all who are interested in FAODs, including patient and parent organizations.

After more than 6 years, including delays caused by the COVID‐19 pandemic, the international guideline is published in this issue of the JIMD, summarizing existing evidence and expert consensus on the treatment of LC‐FAOD. The guideline confirms many established practices as first‐line treatments for these disorders, heavily relying on a rigorous evidence review followed by an extensive Delphi consensus process. Previously, national recommendations based on expert consensus were the principal basis for treatment decisions in long‐chain fatty acid oxidation disorders. Divergent practices across countries were in part due to disparities in the access to medication and diverse regulatory systems governing the classification and reimbursement of dietary therapies, supplements, and pharmacologic agents.

Coordinating international practices allowed harmonization of management, diagnostic, and monitoring procedures across participating countries from Europe, North and South America, and Australasia. However, most recommendations in the guideline were based predominantly on low levels of evidence rather than expert consensus, a common phenomenon in rare disease clinical guidelines, as well as a literature that emphasizes small case series and observational studies rather than large‐scale controlled trials.

## 3 An Important Treatment Goal: Living a Normal and Active Life

In FAODs, as in all inborn metabolic diseases, it is important to address the treatment goal of “living a life as normal as possible.” This includes attending school, participating in sports, and becoming as independent in adolescence as disease allows. As a disorder of energy deficiency triggered by physical stress, patients with LC‐FAOD have often faced restrictions in exercise and physical activity. Increasingly, it is now clear that such restrictions are detrimental in the long term, as older individuals are physically less fit and overweight. Some patients are wheelchair‐bound by adolescence, especially in mitochondrial trifunctional protein (MTP) and isolated long‐chain 3‐hydroxyacyl‐CoA dehydrogenase (LCHAD) deficiencies. In contrast, patients following a formal exercise regimen appear to maintain better physical condition with less muscle pain and weakness. The balance between too much exercise and not enough remains to be defined; however, initial data confirm significant training effects without decompensation from regular exercise, and the concept of exercise prescription has come into focus.

## 4 Remaining Challenges: Unmet Patient Needs and Lack of Clinical Trials

While the LC‐FAOD guideline provides valuable insights into the knowledge gained and progress made over the last 50 years, many treatment‐related challenges remain. Newborn screening programs for FAODs have nearly eliminated neonatal mortality and significantly reduced morbidity, and the identification of early predictive markers of outcome has allowed adjustment of treatment, especially of neonatal regimens. However, functional studies for disease confirmation or severity stratification are not accessible in all countries that perform newborn screening. In general, major concern exists over the need to treat patients with milder defects—with a risk of overtreatment—who are likely to remain asymptomatic for many years. In contrast, some patients have life‐altering and life‐threatening symptoms in spite of early diagnosis and treatment.

Importantly, research has revealed that the pathophysiology of FAODs is more complex than just fat‐derived energy deficiency and metabolite toxicity, with recent studies identifying individual compensatory mechanisms as well as secondary changes in other metabolic pathways, including the tricarboxylic acid cycle and respiratory chain, cardiolipin synthesis, effects on signaling pathways via transcription factors such as peroxisome proliferator activated receptors, and atypical inflammatory reactions related to the accumulation of long‐chain fat derivatives behind the primary metabolic block [3, 4, 9, 10]. These additional derangements require the development of new treatment strategies beyond fasting avoidance or dietary modification. The unique challenges of the broad spectrum of peripheral neuropathy [11] and retinal degeneration in MTP/LCHAD deficiencies [12, 13], in particular, are not sufficiently addressed by current treatment approaches, while recurrent rhabdomyolysis, myopathy, and myalgias are also often not sufficiently controlled in all LC‐FAOD patients.

Treatment of many inborn metabolic disorders has traditionally relied on restricting enzyme substrates and/or replacing the product to restore metabolic balance. For FAODs, it is critical to move beyond this simple concept to a broader understanding of the complex interactions among multiple energy‐producing pathways and secondary pathophysiologic changes, thereby enabling additional targeted therapies that can achieve complete symptom control and improve patient outcomes. The first international guideline on LC‐FAOD outlines current evidence on treatment and identifies gaps in the evidence needed to support rigorous recommendations. As noted earlier, it is glaringly obvious that controlled clinical trials examining most questions are missing. Notably, clinical trials for rare diseases do not necessarily need to be double‐blind, placebo‐controlled, as newer trial designs are increasingly recognized as scientifically rigorous and statistically meaningful. Such trials are likely to play an important role in advancing the FAOD field. Formal evidence is lacking for dosing medium‐chain fat supplements, fasting intervals, and the need for carnitine supplementation; the guideline also leaves these recommendations open. Triheptanoin is approved by the FDA (Food and Drug Administration, US) for the treatment of long‐chain fatty acid oxidation disorders (LC‐FAOD) in the United States, Canada, Brazil, Japan, Mexico, and Kuwait, but is not approved by the EMA (European Medicines Agency) in Europe. A controlled clinical trial is underway in Europe to address the requirements of the EMA.

In addition, a growing population of adult patients with LC‐FAODs requires age‐adapted treatment recommendations for long‐term management, which would benefit from age‐appropriate clinical studies rather than norms transferred from pediatric practice. The challenges of adult topics, such as pregnancy, also need study [14]. Multicenter collaborative studies that enroll all patients in clinical trials as part of routine clinical care would be optimal, as is seen for many cancer therapies. Leveraging larger patient populations will allow multiple studies addressing a variety of clinical questions to proceed concurrently.

Finally, new newborn screening technologies including genomic sequencing [15, 16] will require clear disease confirmation tools and improved early risk assessment for future symptomatic disease [17]. In this respect, a clear algorithm for proceeding after an abnormal genetic or metabolomic newborn screening result must be defined before these new technologies are widely implemented.

## 5 Summary

In the last 50 years, there has been a significant increase in knowledge about LC‐FAOD. Significant improvements in outcomes were due to the implementation of newborn screening programs for LC‐FAOD in many countries worldwide. Despite an improved understanding of pathophysiology, dietetic measures remain the mainstay of treatment. However, personalized approaches based on careful risk assessment have advanced the field and improved outcomes. New treatments for FAODs are in development, including novel anaplerotic agents, ketone body supplementation, and mRNA and gene therapies, and offer hope for further improvements in patient care in the coming years. While extensive effort and discipline were required to finalize this international guideline and reach consensus on harmonized recommendations across countries, a general lack of rigorous clinical evidence should serve as a stimulus to initiate international, multicenter controlled trials. Easily accessible trial designs with flexible research frameworks that use real‐time data to change rules or re‐estimate sizes could be helpful here. In addition, clear disease confirmation tools that allow determination of disease severity need to be easily accessible to define individual treatment requirements for every patient.

Finally, this first international guideline should be considered an evolving document that identifies essential studies to address the significant remaining knowledge gaps as the therapeutic landscape evolves. The development of clinical registries, natural history studies, and collaborative international trials has the potential to close current evidence gaps and contribute to a more evidence‐based future version of this guideline. An international initiative is challenging but also highly rewarding, providing coordinated recommendations worldwide.

## Funding

The authors have nothing to report.

## Conflicts of Interest

U. Spiekerkoetter received grants and consulting fees from Ultragenyx; served as a consultant to the European Metabolic Group (Danone) and the Ultragenyx Advisory board, and received support for meeting attendance from Inform and the European Metabolic Group. The other author declares no conflicts of interest.

## Data Availability

The data that support the findings of this study are available on request from the corresponding author. The data are not publicly available due to privacy or ethical restrictions.
